# Effectiveness of physiological flexion swaddling and oromotor interventions in improving preterm infants' oral feeding ability in the NICU: a randomized controlled trial

**DOI:** 10.1016/j.jped.2024.06.014

**Published:** 2024-09-05

**Authors:** Luh K. Wahyuni, Irawan Mangunatmadja, Risma K. Kaban, Elvie Zulka K. Rachmawati, Rizky K. Wardhani, Budiati Laksmitasari, Boya Nugraha

**Affiliations:** a Faculty of Medicine Universitas Indonesia - Dr. Cipto Mangunkusumo Hospital, Physical Medicine and Rehabilitation Department, Jakarta, Indonesia; b Faculty of Medicine Universitas Indonesia - Dr. Cipto Mangunkusumo Hospital, Department of Child Health, Jakarta, Indonesia; c Faculty of Medicine Universitas Indonesia - Dr. Cipto Mangunkusumo Hospital, Department of Otorhinolaryngology-Head Neck Surgery, Jakarta, Indonesia; d Hannover Medical School, Department of Rehabilitation Medicine, Hannover, Germany

**Keywords:** Oral feeding ability, Oromotor interventions, Physiological flexion swaddling, Preterm infants, NICU

## Abstract

**Objective:**

This study aimed to prove the effectiveness of physiological flexion swaddling and oromotor interventions in terms of the duration needed to achieve the oral feeding ability of preterm infants in the NICU.

**Methods:**

A randomized clinical trial in five Neonatal intensive care units (NICU) was performed involving 70 preterm infants born at 28–34 weeks gestational age. Participants were allocated to 1) the experimental group (n = 39) received physiological flexion swaddling and oromotor interventions, and 2) the control group (n = 31) received conventional swaddling and oromotor interventions. Mann-Whitney U analysis was used to determine the effectiveness of each group according to the duration needed to achieve oral feeding ability, while Kaplan-Meier survival analysis was applied to compare the duration of both groups.

**Results:**

The experimental group had a significantly shorter duration in achieving oral feeding ability [4 (1–15) vs. 7 (2–22) days; p = 0.02]. The Kaplan-Meier survival curve analysis showed that infants in the experimental group achieved full oral feeding ability earlier than those in the control group (15 vs. 22 days).

**Conclusions:**

Physiological flexion swaddling and oromotor interventions have been proven to be effective in shortening the number of days needed to achieve the oral feeding ability of preterm infants in the NICU.

## Introduction

Oral feeding is a complex skill that requires infants to regulate their autonomic, motor, and behavioral states to achieve optimal coordination.^1^ For term infants, oral feeding ability comes naturally; however, it may be considered one of the most challenging tasks for preterm infants because of their underdeveloped oral-motor skills, poor coordination of suck-swallow-breathe, gut immaturity, and other medical issues.^2^^,^^3^ Difficulty transitioning from gavage feeding to oral feeding is also a concern because it often delays the infant's hospital discharge, increases medical costs and parental stress, and potentially leads to feeding-related readmissions and long-term feeding difficulties.^4^, ^5^, ^6^

In clinical practice, it is often found that preterm infants do not have optimal oral feeding ability even though they have been declared ready to feed. The recent study on preterm infants in Indonesia found that self-regulation, physiological flexion postural tone, behavioral state, and levels of morbidity are major factors influencing oral feeding ability.^7^ These factors have not been considered or paid much attention to; hence the author needs to focus on establishing an effective yet safe method to enhance oral feeding ability.

Physiological flexion postural tone is one of the most important factors affecting preterm infants’ oral feeding ability. This includes flexion at the hips, knees, and ankles; rounded shoulders with both hands close to or touching the face; head in the midline; and neck slightly flexed. In most cases, preterm infants lack a normal muscle tone to independently position themselves in physiological flexion; hence they often exhibit a fully extended position.^8^ Prolonged extended position can hinder the acquisition of developmental motor skills and self-regulation and may interfere with oral feeding skills.^9^ Previous studies have indicated that positioning for preterm infants should consider how the fetus experiences a physiological flexed posture in the uterus, which promotes better hand-eye coordination, and self-regulation ability, and saves energy for infants.^9^^,^^10^ Swaddling is one of the numerous ways to maintain physiological flexion in preterm infants; however, its effect on oral feeding ability has not yet been studied.^11^

Another frequent and easily applied technique for enhancing oral feeding ability is oromotor intervention. Oromotor intervention in preterm infants is described as a sensory-motor input to the perioral and intraoral structures, as well as non-nutritive suction of a pacifier to maintain rudimentary oral-motor skills and improve oral muscle tone and movement to facilitate normal oral motor developmental patterns.^6^^,^^12^^,^^13^ Numerous studies have shown that oromotor intervention has beneficial effects on oral feeding in preterm infants such as reduced time of transition from tube feeding to oral feeding, increased feeding efficiency, weight gain, and shortened length of hospital stay, while no negative outcomes have been reported.^2^^,^^12^^,^^14^^,^^15^

Given the importance of the two interventions mentioned, in this study, the authors aimed to combine physiological flexion swaddling and oromotor interventions to enhance oral feeding ability in preterm infants. This study aimed to prove the effectiveness of physiological flexion swaddling and oromotor interventions in terms of the duration needed to achieve oral feeding ability in preterm infants.

## Materials and methods

### Study design

This randomized, controlled, double-blind clinical trial (ClinicalTrials ID: NCT04945967) was conducted in the NICU of Dr. Cipto Mangunkusumo National General Hospital, Harapan Kita Women and Children Hospital, Bunda Women and Children Hospital Menteng, Pasar Rebo Hospital, and Budi Kemuliaan Hospital from August to November 2021. The study was approved by the Research Ethics Committee (protocol number: 21-03-0235). All procedures within this study were performed by trained general practitioners and are applicable by physiatrists, neonatologists, neonatal nurses, and midwives.

### Participants

This study included 70 preterm infants born at 28–34 weeks gestational age who fulfilled the following oral feeding readiness criteria: 1) stable cardiorespiratory status, 2) received full enteral feeding through an orogastric tube for at least 120 mL/kg/day without signs of bloating or vomiting, and 3) strong and rhythmic non-nutritive sucking. The participants fulfilled the three criteria then underwent the objective evaluation of oral feeding ability, which was adapted from the Oral Feeding Skills (OFS) scale by Lau and Smith^16^ as the basis of evaluation.

Using the OFS scale, the authors can measure the rate of milk transfer and feeding proficiency/percentage of volume ingested during the first 5 min of oral feeding to define the endurance and actual feeding ability of the infant while fatigue is deemed minimal. In this study, oral feeding ability was achieved if infants were able to consume > 30% of their prescribed feeding volume during the first 5 min, with a rate of milk transfer of ≥ 1.5 mL/min, and without any signs of aspiration.^16^ Evaluation of oral feeding ability was performed using peristaltic plus nipple for low-birth-weight size SS (Pigeon™, Pigeon Corporation, Tokyo, Japan). Hence, preterm infants who were ready but were not able to feed orally according to the criteria mentioned above were eligible as participants in this study.

Preterm infants with medical complications such as craniomaxillofacial malformations, neonatal asphyxia with a 5-minute APGAR score < 7, grade III or IV intraventricular hemorrhage, and receiving any respiratory support at the time of assessment were excluded from this study. Written informed consent was obtained from the parents or guardians of the infants before their participation in the study.

### Randomization and blinding

Infants meeting the eligibility criteria were randomly assigned to the experimental or control group using a computer-generated randomization sequence placed in sealed opaque, sequentially numbered envelopes. An independent third person not related to the research team was selected by the researcher to randomly assign infants to both groups. Interventions in both groups were double-blinded to ensure no bias.

### Interventions

The experimental group received physiological flexion swaddling and oromotor intervention which included oral stimulation, three-finger jaw control stimulation, and non-nutritive sucking stimulation with a preemie pacifier; whereas the control group received conventional swaddling technique and oromotor intervention which included oral stimulation and non-nutritive-sucking stimulation with a usual pacifier in the NICU. Interventions in both groups were administered by general practitioners who had been given training beforehand. Interventions were given once a day, 30 min before the infant's gavage feeding schedule, on the infant radiant warmer. Both groups also received routine feeding and kangaroo mother care (KMC), as per the protocol. Interventions were done until oral feeding ability achievement and further evaluation is to be made after hospital discharge.

### Experimental group

The first step of the intervention in the experimental group was physiological flexion swaddling. This swaddling technique aims to provide external stabilization to preterm infants who have not been able to perform internal stabilization, to help the infant achieve body alignment in order to facilitate movement towards the midline. This swaddling technique also ensures that the infant's neck is positioned on the midline; shoulders protracted and adducted, elbows flexed, hands toward face or mouth, ‘C’ alignment on the vertebrae, hip flexed and tilted posteriorly, and knee flexed with 90° popliteal angle.

**Figure 1:**
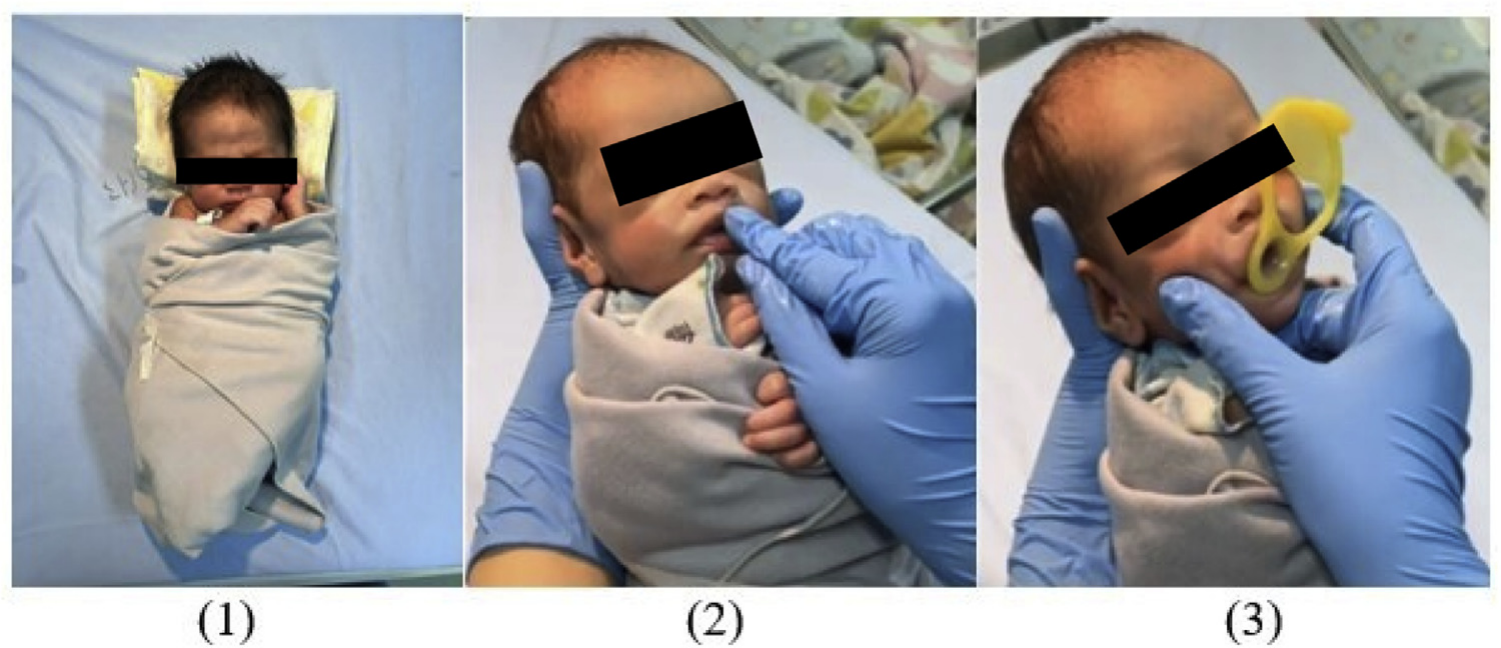
Series of Interventions Given in the Experimental Group. Step 1: Physiological flexion swaddling. Step 2 & 3: Oromotor interventions: Oral stimulation, three-finger jaw control, and non-nutritive sucking stimulation using a premiee pacifier.

After the infant was swaddled accordingly, oromotor interventions including oral stimulation, three-finger jaw control, and non-nutritive sucking stimulation using a preemie pacifier (Pigeon™, Pigeon Corporation, Tokyo, Japan) were performed. Steps of oral stimulation were given according to the Fucile protocol^17^ and lasted for 15 min. Three-finger jaw control is a proprioceptive stimulation consisting of simultaneous movement, jaw elevation, and depression to help infants form negative intraoral pressure. In this study, three-finger jaw control and non-nutritive sucking stimulation using a preemie pacifier were performed simultaneously. In total, the series of interventions in the experimental group took approximately 20–30 minutes and its steps are shown in Figure 1.

### Control group

A series of interventions in the control group included conventional swaddling, followed by oral stimulation based on the Fucile protocol^17^ for approximately 15 min, including non-nutritive sucking stimulation using a usual pacifier in the NICU. In total, the series of interventions in the control group took 20–25 minutes. After each intervention session, oral feeding ability parameters were re-evaluated using the OFS scale. Interventions in both groups were discontinued if the infants achieved oral feeding ability. The programs were interrupted if the infants were medically unstable (episodes of desaturation, apnea, tachycardia and bradycardia) during the intervention or evaluation phase. Infants were dropped out if they experienced medical instability during three consecutive sessions.

### Statistical analysis

Sample size calculation in this study was determined by the clinical superiority design formula, resulting in a minimum of 16 participants for each study group, a 20% dropout estimation was then added. Participants’ baseline characteristics were analyzed using an independent *t*-test or Mann-Whitney U test for continuous variables and chi-square or Fisher's exact test for categorical variables. The Mann-Whitney U test was used to compare the effectiveness of the experimental versus control on the duration needed to achieve oral feeding ability and was expressed as median and range (days). Kaplan-Meier survival analysis was used to compare the duration needed to achieve oral feeding ability in both groups. Statistical analysis was performed using the intention-to-treat principle and statistical significance was set at p < 0.05. SPSS statistical software version 20.0 (IBM, SPSS) was used to analyze the data.

## Results

**Figure 2:**
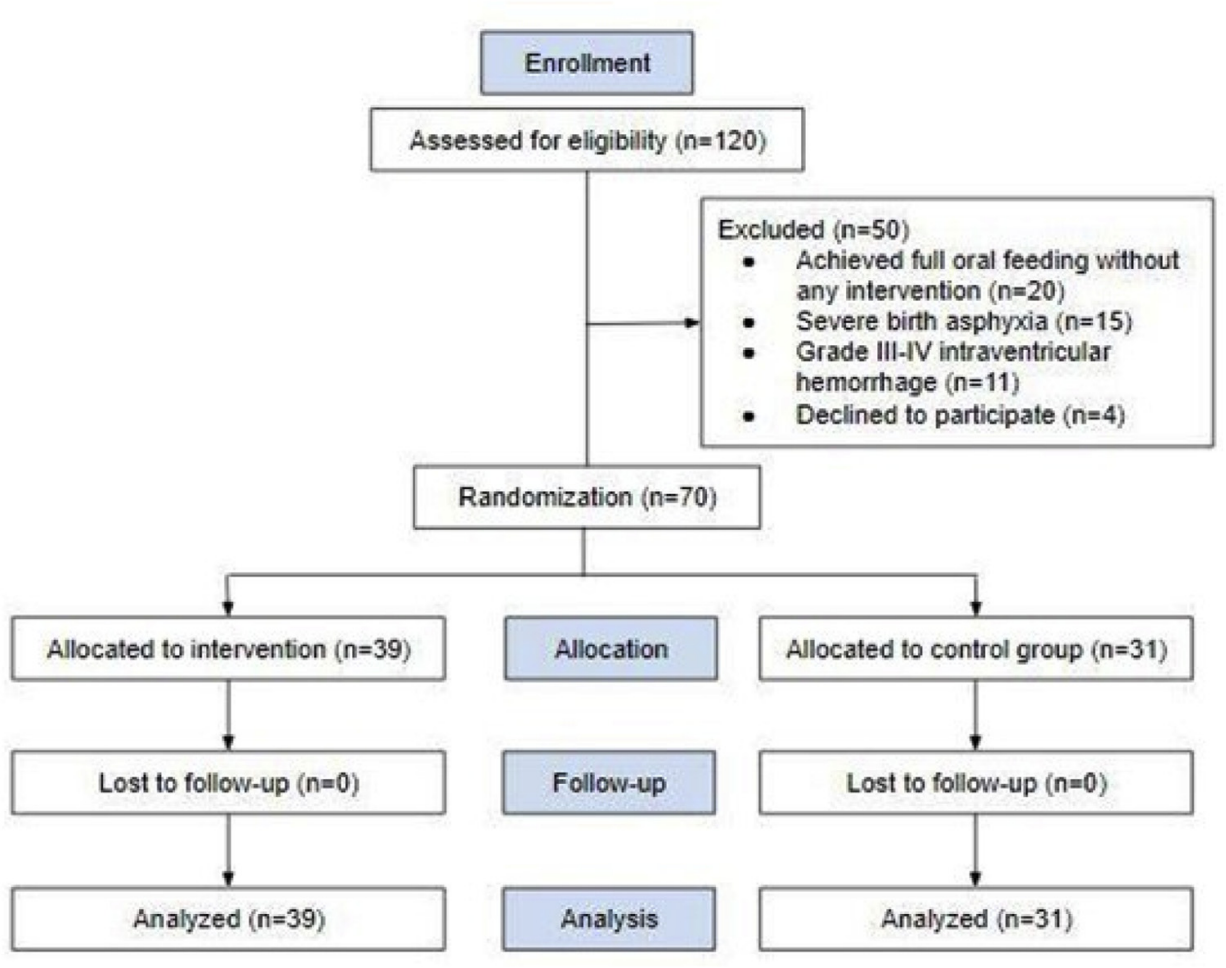
Flow of study participants.

A total of 120 preterm infants were screened for eligibility, and 70 preterm infants were eligible for randomization and analysis with no instances of dropout. Most eligible infants were 32–34 weeks PMA, therefore interventions begin from 32 weeks PMA. Fifty infants were excluded because of unmet inclusion criteria (20 had achieved full oral feeding ability before any intervention, 15 had severe birth asphyxia, 11 presented grade III–IV intraventricular hemorrhage, and four declined to participate). The allocation of subjects was not equal between the two groups (39 vs. 31) (Figure 2). This minimizes any bias by eliminating predictability while still considering an equal proportion in simple random sampling, being within a ratio difference of 45–55%.^18^ Furthermore, each subject maintained complete randomness with regard to the treatment administered. The participants in both groups had statistically similar baseline characteristics (p > 0.05) (Table 1).

**Table 1:** Baseline characteristics of the study population.

Characteristics	Experimental group (n = 39)	Control group (n = 31)	p value
	n (%)	n (%)	
**Gender**			
Male	19 (48.7)	11 (35.5)	0.266
Female	20 (51.3)	20 (64.5)	
**Gestational age (weeks)**			
28–31	20 (51.3)	11 (35.5)	0.186
32–34	19 (48.7)	20 (64.5)	
**Postmenstrual age (weeks)**			
32–34	19 (48.7)	19 (61.3)	0.548
35–36	16 (41)	9 (29)	
37–42	4 (10.3)	3 (9.7)	
**Birth weight**			
ELBW	3 (75)	1 (25)	0.691
VLBW	17 (56.7)	13 (43.3)	
LBW	19 (52.8)	17 (47.2)	
**Self-regulation**			
Able	29 (52.7)	26 (47.3)	0.335
Unable	10 (66.7)	5 (33.3)	
**Postural tone**			
Inadequate	15 (45.5)	18 (54.5)	0.103
Adequate	24 (64.9)	13 (35.1)	
**Behavioral state**			
Not alert	25 (51)	24 (49)	0.227
Alert	14 (66.7)	7 (33.3)	
**Physiological stability**			
Stable	16 (51.6)	15 (48.4)	0.538
Unstable	23 (59)	16 (41)	
**Morbidity (NMI)**			
High (NMI IV–V)	10 (55.6)	8 (44.4)	0.877^a^
Moderate (NMI III)	20 (57.2)	15 (42.9)	0.775^b^
Low (NMI I–II)^#^	9 (52.9)	8 (47.1)	

NMI, Neonatal Medical Index; Low morbidity was used as a reference.

a p value of high morbidity towards low morbidity.

b p value of moderate morbidity towards low morbidity.

**Figure 3:**
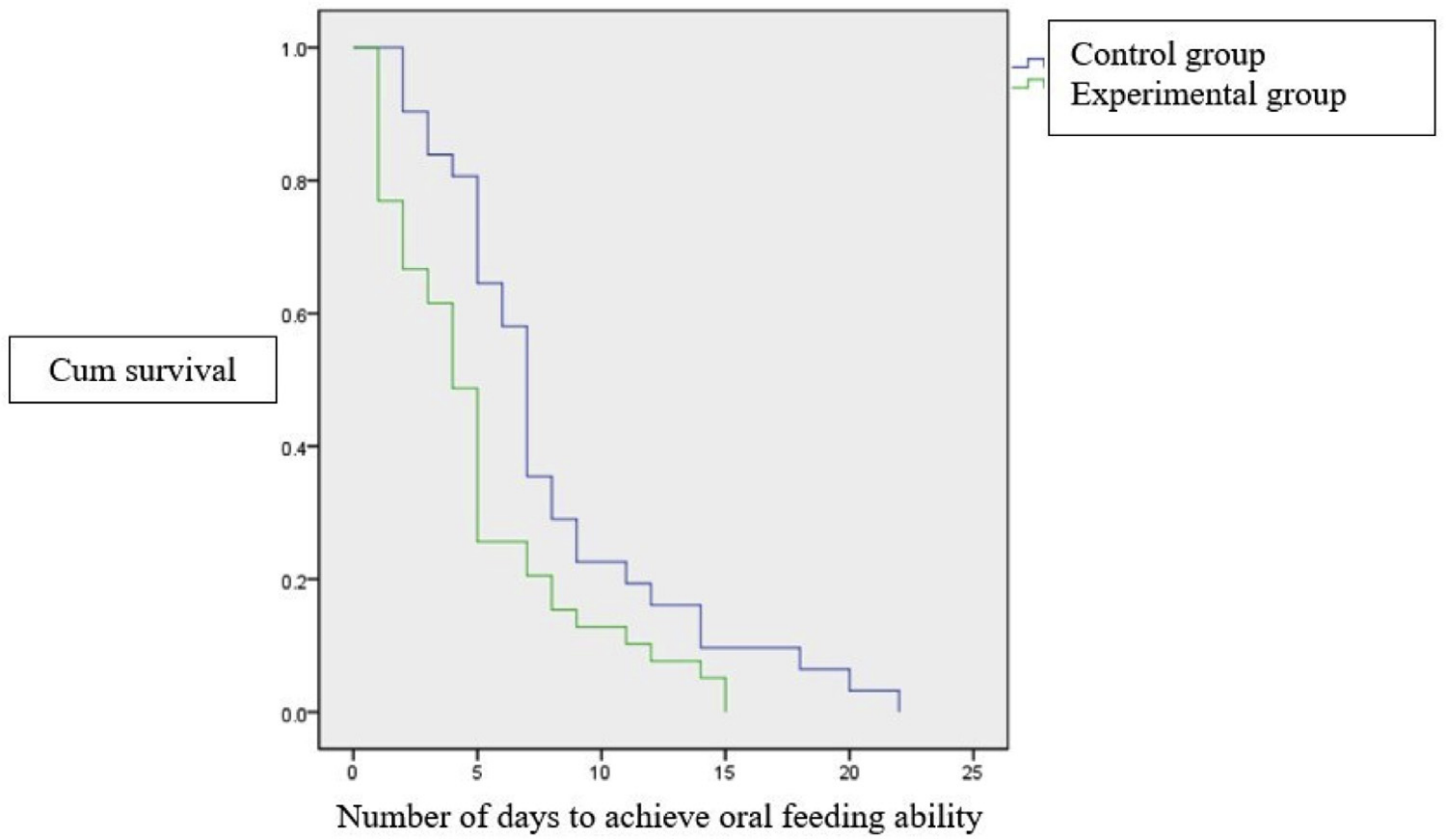
Kaplan-Meier survival curve showing number of days to achieve oral feeding ability.

Compared to the control group, the experimental group had a significantly shorter duration to reach oral feeding ability with a median value of 4 (1–15) days, compared to the control group 7 (2–22) days, with p = 0.02. Based on the Kaplan-Meier survival curve analysis, all subjects in the experimental group achieved full oral feeding ability in 15 days, whereas the control group reached full oral feeding for at least 22 days (Figure 3). The experimental group was found to be 1.46 times more effective in managing oral feeding ability in preterm infants. No adverse effects were observed in either group.

## Discussion

The results of this study showed that the series of interventions given in the experimental group (physiological flexion swaddling, oromotor interventions that included oral motor stimulation, three-finger jaw control, and non-nutritive sucking stimulation using a preemie pacifier) were associated with a significantly shorter number of days to achieve oral feeding ability than the control group (4 vs. 7 days; p = 0.02). The shorter number of days to achieve oral feeding ability in the preterm infants in the experimental group was based on various factors.

The combination of physiological flexion swaddling and oromotor interventions in the form of oral stimulation, three-finger jaw control, and non-nutritive sucking stimulation using a preemie care pacifier is a comprehensive series of interventions that cannot be separated from each other. Each component in this intervention was designed as a problem-solving solution to the factors that are significantly related to oral feeding ability in preterm infants, namely self-regulation, postural tone, and behavioral state, obtained from the results of previous cross-sectional study.^7^ This series of interventions also provided opportunities for infants to learn and develop the ability and strength to perform functional sucking movements with optimal movement patterns, suck-swallow-breathing coordination, and protect the airway from the risk of aspiration.

In the experimental group, the postural tone of preterm infants’ and its influence on various body functions have received serious attention. Swaddling aims to provide external stabilization to preterm infants to maintain postural tone during the feeding process. A stable trunk and pelvis during physiological flexion swaddling enabled the infant to control his distal body parts, such as the neck, shoulder girdle, jaw, lips, cheeks, and tongue. Infants who already have full control of their motor will find it easier to coordinate the suck-swallow-breath process during the feeding process.^19^ Stable physiological flexion optimizes the oromotor muscles for suck-and-breathe coordination. In addition, swaddling using a cloth that covers the infant's entire body in a physiological flexion position will build a perception similar to the condition in the mother's womb, and the whole body is stabilized by the uterine wall to prevent random movements or uncontrolled motor activity. Infants who feel safe and calm can save their energy, which in turn builds good self-regulation skills.^20^^,^^21^

Oromotor intervention was based on the philosophy of neuroplasticity related to stimulation and the dynamic system theory of motor control and motor learning**.** In 2019, Maier et al.^22^ stated that there are 15 principles of brain plasticity eight of which are relevant to the principle of oral stimulation, namely: goal-oriented practice, social interaction, multisensory stimulation, modulate effector selection, task-specific practice, massed/repetitive practice, spaced practice, and dosage/duration. The application of the principle of brain plasticity was that oral stimulation has a clear goal to improve infants’ oral feeding ability; interventions were carried out in a structured manner by medical personnel, a series of specific multisensory stimulations in the form of tactile and proprioceptive stimuli through synergistic movements of three-finger jaw control, as well as the use of pacifiers given according to the trigeminal, facial, and glossopharyngeal nerve receptors which were integrated at the level of the central nervous system (reticular formation, limbic system, and CPG) to produce responses through the trigeminal, facial, glossopharungeal, vagal, and hypoglossal nerves in the form of rooting reflex, sucking movement (movement pattern, strength, resistance), suck-swallow-breathing coordination, arousal, self-regulation ability, and behavioral state. Stimulation was carried out once a day, 20–30 minutes before the feeding schedule. Based on the dynamic system theory regarding motor control and motor learning, preterm infants’ success in achieving oral feeding ability was the outcome of the interaction of three main factors: preterm infant (physiological flexion postural tone, behavioral state, self-regulation, and morbidity), activity (sucking movements, suck-swallow-breath coordination, and airway protection) performed by the infant, as well as the role of the medical and paramedical teams in providing adequate stimulation (therapeutic positioning and oromotor intervention) in achieving functional abilities. Preterm infants who have weak sucking, inefficient feeding, and are tired easily develop strength, endurance, and sucking skills, as well as coordination of the suck-swallow-breathing process through oromotor intervention.^22^^,^^23^

The three-finger jaw control stimulation in this study aims to support the stability of the cheek and jaw areas, help coordinate swallowing, and stimulate the lip seal, thus minimizing the volume that leaks during feeding. Cheek and jaw stability facilitates control of the tongue and movement improves the efficiency of the sucking process.^14^^,^^24^ This statement was supported by Hwang,^25^ who found feeding rate in the first 5 minutes was faster, (3.16 vs. 2.74 mL/min; p = 0.046) and a less average percentage of leakage while feeding (5.17% vs. 7.27%; p = 0.040) in the group of preterm infants who received oral support compared to the control group.

Non-nutritive sucking stimulation using a preemie pacifier was based on the size and shape of the lips, as well as the size of the oral cavity of preterm infants. This application of the pacifier supports the goodness-of-fit principle which states that the more appropriate the size of the pacifier to the oral cavity, the less effort is required to perform a lip seal and form negative pressure to suck.^19^ A study by Fucile^26^ showed that providing appropriate non-nutritive sucking stimulation increased sucking strength and endurance, improved behavioral state, improved coordination of suck-swallow-breathing, and shortened the duration of oral feeding. This statement was also supported by Zhang et al.^27^ who reported that non-nutritive sucking stimulation in preterm infants using pacifiers was proven to shorten the average number of days to achieve oral feeding ability, compared to the control group (10.0 vs. 14.6 days; p < 0.001).

This study contributes to the medical teams involved in the management of premature infants: 1) a series of interventions consisting of physiological flexion swaddling and oromotor interventions which were performed once a day, took approximately 20–25 minutes, was a relatively easy therapeutic approach, did not require any expensive equipment or] long duration, and had no side effects. Hence, it can be used as an option to improve oral feeding ability in preterm infants, 2) Swaddling, which seems to be a simple procedure, has become a part of daily management. However, swaddling has to be performed in the correct procedures as it will affect various body functions, 3) pacifiers given to preterm infants must be viewed as a medical device, similar to other medical instruments because it possesses therapeutic objectives to stimulate non-nutritive sucking ability, provide a sense of security, improve behavioral state and self-regulation, and 4) the size and shape of the preemie pacifiers should fit the oral cavity of preterm infants in order to minimize the force required for the infant to create negative intraoral pressure.

## Limitations

The present study did not assess the infants’ endurance during feeding, as it may or may have not affected their oral feeding ability. Other non-significant trends found in this study were uneven between groups since the author did not consider them as the focus of this study, but it may be considered for future studies.

## Conclusions

Physiological flexion swaddling and oromotor interventions have been proven to be effective in shortening the number of days needed to achieve the oral feeding ability of preterm infants in the NICU.

## Authors’ contributions

Conception and design of the study: LKW, IM, RKK, EZK; Statistical analysis: RKW, BL; Drafting the article: LKW, RKK, BL; Revising critically for important intellectual content: LKW, IM, RKK, EZK, BN. All authors read and approved the final manuscript.

## Data availability

The data associated with the paper are not publicly available but are available from the corresponding author on reasonable request.

## Conflicts of interest

The authors certify that there is no conflict of interest with any financial organization regarding the material discussed in the manuscript.
